# Determine the structure of phosphorylated modification of icariin and its antiviral activity against duck hepatitis virus A

**DOI:** 10.1186/s12917-015-0459-9

**Published:** 2015-08-14

**Authors:** Wen Xiong, Xia Ma, Yi Wu, Yun Chen, Ling Zeng, Jiaguo Liu, Weidong Sun, Deyun Wang, Yuanliang Hu

**Affiliations:** Institute of Traditional Chinese Veterinary Medicine, College of Veterinary Medicine, Nanjing Agricultural University, Nanjing, 210095 People’s Republic of China; Pharmaceutical Engineering Department, Henan University of Animal Husbandry and Economy, Zhengzhou, 450011 People’s Republic of China

**Keywords:** Icariin, Phosphorylated structural modification, HRESIMS, NMR, Duck hepatitis virus A

## Abstract

**Background:**

Our previous research showed that icariin (**1**) and its phosphorylated structural modification (**2**) improved the survival and attenuated oxidative stress and liver dysfunction induced by duck virus hepatitis. In this paper, we were one step closer to determine the structure of phosphorylation icariin (**2**) by the FT-IR, HRESIMS and ^13^C NMR. Anti-DHAV activities of **1** and **2** were compared in duck embryonic hepatocytes (DEHs) cultured *in vitro* and by artificial infection method *in vivo*. Additionally, the antiviral mechanisms of replication/release *in vitro* and the DHAV gene expression *in vivo* of **1** and **2** were analyzed.

**Results:**

Compound **2**'s molecular formula was C_33_H_42_O_18_P. The results indicated that **1** and **2** effectively resisted DHAV invading DEHs, that they decreased the mortality of ducklings challenged with DHAV, and that **2** performed more effectively. **1** and **2** performed evenly on DHAV release; however, **2** restrained virus replication far more effectively. Since the anti-DHAV mechanisms of **1** and **2***in vitro* probably involve suppression of replication and release, **2**’s better performance in anti-DHAV may result from its far more effectively inhibiting virus replication.

**Conclusions:**

The compound **2**'s chemical structure was defined as 8-prenylkaempferol-4'-methylether-3-rhamnosyl-7-(6'''-phosphate)-glycoside. **1** and **2** exhibited anti-virus activity on DHAV. Our results suggest that **1** and **2** might become an anti-virus plant material candidate.

## Background

Duck viral hepatitis (DVH) is an acute, contagious, highly fatal disease of young ducklings, which was first described on Long Island, NY, USA, in 1949 [1]. The causative agent, duck hepatitis virus (DHV), historically belongs to the family *Picornaviridae* and is divided into three serotypes (DHV-1, 2, and 3). DHV-1 is the most widely distributed and the most virulent one among the three types of DHV [2]. Recently, sequence analysis of duck hepatitis virus type 1 led to its classification as the only member of a new genus, *Avihepatovirus*, of the family *Picornaviridae*, so the DHV-1 was renamed to DHAV [3]. DHAV predominantly infects young ducklings aged less than three weeks. Clinically, the disease is acute, rapidly spreading, with a shorter course and high mortality rate. Meanwhile, the DVH is an attractive research model of human hepatitis, with great potentialities in both human and veterinary medicine [4]. In the absence of effective anti-DHAV drugs, immunizing ducks or young ducklings with attenuated DHAV vaccines is the primary approach to disease control. However, vaccines are not totally effective and any clinical manifestation may cause potentially irreparable damage.

**Fig. 1:**
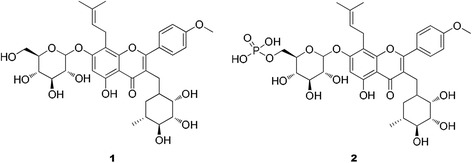
Structure of icariin (1) and 6'''-phosphate icariin (2)

Several types of flavonoids including those from epimedium and propolis as well as prescriptions of flavonoids ingredients showed significant resistance to Newcastle disease, infectious bursal disease and other viral diseases in our previous experiments [5], although the underlying mechanisms were not researched in detail. *Epimedium* is a well-known traditional herbal medicine in China used for centuries to invigorate kidney and strengthen kidney – *yang*. Contemporary pharmacologic experiments indicate that Epimedium flavone has anti-aging, anti-tumor and anti-osteoporosis activities [6–9]. Icariin (C_33_H_40_O_15_, **1**, Fig. 1.) is the main effective component of *Epimedium brevicornum Maxim* [10]. **1** exhibits many biological activities and pharmacological effects, including antioxidant, anti-inflammatory, anti-tumor [11]. Previous research showed that **1** enhanced the cellular and humoral immunity in mice, and increased the phagocytic activity of macrophages in immune-depression mouse models caused by massive doses of hydrocortisone and hydroxycarbamide [12]. Therefore, we choose the **1** as the study object.

Molecular modification of flavonoids and polysaccharide became an important research field along with the increasing pursuit about manifold biological activities of modified. Many studies confirmed that the biological activities of the Chinese herbal ingredients could be improved by the chemical method (sulfated, phosphated, selenylation). In our previous published work, we found that the compound **1** and phosphorylated icariin (C_33_H_42_O_18_P, **2**, Fig. 1) could alleviate the oxidative stress and liver injury in ducklings caused by DHAV. These outcomes were better in ducklings treated with **2** than in those treated by **1**. In clinical trials, compared with **1**, **2** could significantly improve survival rate [13]. This is the most important to the meaning of the actual production application. So we believe that this novel structure resulted in the difference. But between the **1** and **2**, their antioxidant effect was similar. It’s suggested that there may be another resistance mechanisms to anti-DHAV infection. To further study its resistance mechanism, we hold the opinion that it is essential to confirm the structure of **2** and study the effect of drug for viral multiplication process. Above on, we determined the chemical structure of **2** by the FT-IR, HRESIMS and ^13^C NMR and the compound **1** and compound **2** antiviral efficacies against DHAV *in vivo* and *in vitro* were investigated.

## Methods

### Reagents and virus

Dulbecco’s modified eagle medium (DMEM) (Gibco) supplemented with streptomycin 100 IU/mL, penicillin 100 IU/mL, 10% fetal bovine serum and glutamine 0.75 mg/mL was used as the nutritive medium. The fetal bovine concentration of the maintain medium (MM) was reduced to 1 %. Dulbecco’s Hanks Balanced Salt Solution (D-Hank’s) was used for washing the embryo. The pH of D-Hank’s, DMEM and MM solutions was adjusted to 7.4 with 5.6 % NaHCO_3_ and stored at 4 °C. Trypsin (Amresco) was dissolved with D-Hank’s up to 0.2 %. The 3-(4,5-dimethylthiazol-2-yl)-2,5-diphenyltetrazolium bromide (MTT, Amresco) was dissolved to 5 mg/mL with phosphate-buffered saline (PBS), filtered through a 0.22 μm syringe filter, and stored at 4 °C in dark bottles. Other chemicals used in the experiment were analytical grade. RNAiso Plus Reagent (Lot no. 9108), PrimeScript RT Master Mix Kit (Lot no. RR036A) and SYBR Premix Ex Taq (Tli RNaseH Plus) Kit (Lot no. RR036A) were bought from Takara. Fluorescein isothiocyanate-labeled rabbit anti-DHAV (Lot no. orb8860) was bought from biorbyt. DHAV (LD_50_:5 × 10^−3^) strain *LQ*_*2*_ used in the challenge experiments was supplied by the Shandong Institute of Poultry (Shandong, China,−70 °C storage). All other chemicals are from standard commercial suppliers, having analytical grade quality.

### Preparation of icariin

Compound **1** was purified repeatedly from the extract of *Epimedium segittatum* by column chromatography (CC) and prepared HPLC. The dried leaves (5 kg) of this plant were extracted three times with 85 % (v/v) EtOH under refluxed (40 L each time for 2 h). The combined extract was concentrated under reduced pressure to give a residue (0.3 kg), which was further partitioned with petroleum ether (PE, boiling point 60–90 °C, 4 L), ethyl acetate (EtOAc, 4 L) and *n*-BuOH (4 L), successively. The EtOAc fraction (38 g) was subjected to a silica gel CC (400 g, 100–200 mesh, 600 × 40 mm) eluted with a step gradient of CH_2_Cl_2_-MeOH-H_2_O (90:1:0.1, 40:1:0.1, 20:1:0.1, 10:1:0.1, 5:1:0.1, v/v, 3 L each step gradient) to give five sub-fractions (E1-E5, 2.2 g, 3.1 g, 3.8 g, 4.6 g, 8.9 g, respectively). E4 (0.2 g) was then purified by Sephadex LH-20 CC (1200 × 24 mm) with MeOH-H_2_O (2:1 to 1:1, v/v, 1.2 L each gradient) to obtain crude flavonoids (0.12 g). The crude flavonoids were further separated by prepared HPLC (column Kromasil 250 × 10 mm, 5 μm, CH_3_CN-H_2_O, 23:77, v/v, 1.6 L) and recrystallized with water repeatedly to yield Icariin (83 mg), whose purity quotient was over 98 %.

HPLC conditions: YMC-Triart C18, (5 μm, 4.6 × 250 mm; YMC), solvent system: A - MeOH, B-H_2_O, C - MeCN, D - 0.2 % H_3_PO_4_; flow rate: 1 mL/min; injection volume: 20 μL; Sample concentration: 10 mg/mL in MeOH. DAD conditions: 270 nm.

HRESIMS conditions: positive ion mode; scan range: 500–4000 *m/z*; source temperature: 300 °C; ion spray voltage 4 kV.

### Preparation of phosphorylated icariin

2.5 g sodium trimetaphosphate and 1.0 g sodium tripolyphosphate were mixed in 50 mL of distilled water, with stirring. Compound **1** (500 mg) was dissolved in 100 mL of distilled water and added to the sodium trimetaphosphate-sodium tripolyphosphate reagent, and stirred in a water bath at 70 °C for 6 h at pH 9. The resulting solution was dialyzed, purified by ODS column chromatography and preparative HPLC (YMC, 4 μm, 150 mm × 20 mm, MeCN/H_2_O, 35: 65), UV detection at 210 nm, and lyophilized to yield compound **2**.

Icariin (**1**): yellow amorphous powder; FT-IR (KBr) ν_max_:3396,1651,1600,1500,1453 cm^−1^;^13^C NMR (DMSO-d_6_, 100 MHz) data in Table 1; HRESIMS *m/z* 676.7400 [M + H]^+^(calcd for C_33_ H_41_ O_15_, 677.2396).

**Table 1:** ^13^C NMR (100 MHz) data of Compounds 1 − 2^a^

	**1** ^b^	**2** ^b^
position	δ_C_,type	δ_C_,type
2	157.80,C	157.79,C
3	135.11,C	135.12,C
4	178.77,C	178.77,C
5	160.98,C	160.99,C
6	98.59,CH	98.60,CH
7	161.88,C	161.89,C
8	108.77,C	108.78,C
9	153.48,C	153.49,C
10	106.06,C	106.07,C
11	21.89,CH_2_	21.90,CH_2_
12	122.60,CH	122.61,CH
13	131.58,C	131.58,C
14	25.94,CH_3_	25.93,CH_3_
15	18.33,CH_3_	18.33,CH_3_
1'	122.73,C	122.74,C
2',6'	131.04,CH	131.04,CH
3',5'	114.55,CH	114.56,CH
4'	159.56,C	159.56,C
1''	102.46,CH	102.46,CH
2''	70.12,CH	70.13,CH
3''	70.55,CH	70.55,CH
4''	71.18,CH	71.17,CH
5''	70.78,CH	70.79,CH
6''	17.93,CH_3_	17.93,CH_3_
1'''	101.01,CH	101.01,CH
2'''	73.83,CH	73.84,CH
3'''	77.07,CH	77.08,CH
4'''	71.58,CH	71.85,CH
5'''	77.66,CH	77.67,CH
6'''	61.10,CH_2_	70.17,CH_2_
OCH_3_	55.98,CH_3_	55.99,CH_3_

^a^The chemical shifts (δ) are expressed in parts per million. ^b^Data recorded in dimethyl sulfoxide-d_6_

Phosphorylated icariin (**2**): yellow amorphous powder; FT-IR(KBr) ν_max_:3384,1652,1610,1430,1504,1148,919 cm^−1^; ^13^C NMR (DMSO-d_6_, 100 MHz) data in Table 1; HRESIMS *m/z* 757.2034 [M + H]^+^(calcd for C_33_ H_42_ O_18_ P, 757.2072).

### Animal experiments

Animal experiments conformed to the Guide for the Care and Use of Laboratory Animals published by the US National Institutes of Health (NIH Publication, Eighth edition, 2011) and were approved by the Nanjing Agricultural University Animal Care Committee (No. 20130093, 2013). To ameliorate suffering, animals which were not expected to survive were humanely euthanized. All steps were complied with AVMA Guidelines for the Euthanasia of Animals (2013 Edition).

One-day-old cherry valley ducks were purchased from Nanjing Tangquan Poultry Farm (Nanjing, China). Ducks were housed in wire cages (60 cm × 100 cm) in air conditioned rooms at 37 °C with lights on for 24 h before the study. The temperature was gradually reduced to room temperature and 12-h light/12-h dark phases, which were kept constant for the remainder of the study. Ducks were fed a commercial starter diet provided by the feed factory of Jiangsu Academy of Agricultural Science (Nanjing, China). Three-day-old cherry valley ducks (n = 180) were randomly divided into four groups: **1**-treated group, **2**-treated group, virus control (VC) group, and a blank control (BC) group (separately reared). Ducklings allocated to the **1**, **2** and VC groups were intramuscularly injected with 0.2 mL of DHAV (20 × LD_50_) per duckling. Ducklings allocated to the **1** or **2** groups were given aqueous **1** or **2** at the dosage of 31.25 mg/Kg (net concentration) of per duckling, *SID*, for 3 days starting on the same day as DHAV injection. Before used, **1** was prepared with 3 % ethanol dissolved, and added a co-solvent 0.5 % Tween 80. In order to ensure the consistency of test, we added the equal amount of ethanol and Tween 80 in the drinking in the **2**, VC and BC groups. The dissolution characteristic of **2** is instant into the water. Blood samples were taken from five ducklings in each group at the 4^th^h, the 8^th^h, and the 54^th^h after injecting DHAV. Half of each blood sample was treated with heparin for anticoagulation and the remainder was left to coagulate. Number of ducks which had been taken blood samples needed to eliminate (n = 15 per group).

### Preparation of duck embryonic hepatocytes (DEHs) and DHAV

Livers were removed from 14-to-16-day-old duck embryos in sterile conditions followed by gallbladder evisceration [14]. The livers were washed three times with D-Hank’s. The tissue was minced with eye scissors and washed three times with D-Hank’s. Liver tissue was digested with a solution of 0.2 % trypsin at 37 °C for 4 to 6 min. As soon as the trypsin was completely absorbed, the tissue was rinsed three times with D-Hank’s and an appropriate amount of DMEM growth medium was added. The seeding cell density was controlled within the range of 0.8 × 10^6^-1.2 × 10^6^ cell/mL, incubated at 37 °C in a humid atmosphere of 5 % CO_2_, the growth medium replaced after 24 h [15]. DMEM was removed when the duck embryo hepatocytes grew into monolayer. The duck embryonic hepatocytes monolayer (DEHs) was washed with calcium and magnesium-free phosphate-buffered saline (CMF-PBS, pH 7.4) once, and left as standby until CMF-PBS was removed again. The DHAV used for challenge experiments and antiviral assays was supplied by the Shandong Institute of Poultry in China and proliferated by inoculating DEHs. The TCID_50_ of the virus liquid was 1 × 10^−3^ by Reed-Muench assay [16]. It was diluted into 5 × 10^−2^(50 TCID_50_) with MM and used for antiviral assays.

### Antiviral activity *in vitro*

The maximum safe concentration of **1** was 250 μg/mL tested by preliminary experiment with MTT method while that of **2** was 1250 μg/mL. **1** was serially twofold diluted with MM from 250 μg/mL to 31.25 μg/mL, and **2** from 1250 μg/mL to 156.25 μg/mL, thus obtaining 4 concentrations each. 70 μL DHAV was added to each well of 96-well culture plate containing the DEHs monolayer. Meanwhile, cell control (CC) group and virus control (VC) group were set. The plate was cultured at 37 °C in a humid atmosphere of 5 % CO_2_ for 2 h. The virus solution was removed and the monolayer was washed three times with D-Hank’s. Subsequently, 70 μL dilution of test ingredient was added to test well using 5 wells per concentration. The plate was left at 37 °C in a humid atmosphere of 5 % CO_2_. As the VC group showed significantly cytopathic effect (approximately after 72 h), the MTT method was used to determine the DEHs activity [17]. The A_570_ value and virus inhibitory rate [(Ā_drug+virus_ − Ā_virus control_)/(Ā_cell control_ − Ā_virus control_) × 100 %] were considered as the indicators of antiviral activity *in vitro* [18].

### Quantitative analysis of blood DHAV

To monitor the blood virus contents in virusemia phases (the 4^th^h and the 8^th^h) and stable (the 54^th^h) phases of the disease, blood samples randomly taken from 5 feathers per group at the 4^th^h, the 8^th^h and the 54^th^h after challenged with virus were collected, treated with heparin anticoagulation and used for testing its virus nucleic acid contents. Real-time polymerase chain reaction (Real-time PCR) was employed in the quantitative analysis of DHAV gene expression at the 4^th^h, the 8^th^h and the 54^th^h *in vivo* [19].

### Clinical effect

Mortality was calculated after all the young ducklings ceased to die using the equation: Mortality of each group (%) = Death in each group/effective samples in each group × 100 %, and the number of ducks providing blood samples needed to be eliminated (15 feathers per group).

### Analysis of virus replication

400 μL DHAV was added into the 24-well culture plate containing a DEHs monolayer, while the CC and VC wells were reserved. The plate was incubated at 37 °C for 2 h in a humid atmosphere of 5 % CO_2_, and the virus solution removed. The plate was washed 3 times with D-Hank’s. Later, 400 μL dilution of test drug was added into the test wells, three wells in parallel, as the most effective antiviral concentration. The 24-well culture plate was incubated at 37 °C for 24 h in a humid atmosphere of 5 % CO_2_ and the total RNA was extracted. Real-time PCR was used to analyze the extent of virus replication.

### Analysis of virus release

400 μL DHAV was added into the 24-well culture plate containing monolayer DEHS, while the CC and VC wells were reserved. The plate was incubated at 37 °C for 2 h in a humid atmosphere of 5 % CO_2_. The virus solution was removed and the plate was washed 3 times with D-Hank’s. 400 μL dilution of test drug at the most effective antiviral concentration was then added into the test wells, three wells in parallel. The plate was continuously incubated at 37 °C in a humid atmosphere of 5 % CO_2_ for 48 h. The supernatant of cells was centrifuged and the sediment was removed. 100 μL supernatant and 100 μL DEHs (0.8 × 10^6^-1.2 × 10^6^ cells/mL, as the internal reference) were mixed together and the total RNA was extracted. Finally real-time PCR was used to analyze the virus release content.

### Primer design

The primers based on the complete genome of DHAV (ZJ strain; Genbank: EU841005) were designed using Primer Premier Software (version 5.0). The forward and reverse primers were 5′-GCCACCCTTCCTGAGTTTGT-3′ (positions: 3336–3355) and 5′-TACCATTCCACTTCTCCTGCTT-3′ (positions: 3489–3510), respectively. Duck Beta-actin was selected as the control. The primers based on the complete coding sequence of *Anasplatyrhynchos* beta-actin mRNA (Genbank: EF667345) were designed using Primer Software (version 5.0). The forward and reverse primers were 5′-CTTTCTTGGGTATGGAGTCCTG-3′ (positions: 826–847) and 5′-TGATTTTCATCGTGCTGGGT-3′ (positions: 995–1014), respectively.

### Statistical analysis

2^-ΔΔCT^ method was used to analyze relative gene expression data [20]. The data of A_570_ value and relative gene expression value were expressed as the mean ± S.D.. Duncan’s multiple range tests were used to analyze the difference among groups with the software SPSS 20.0. *χ*^*2*^-test was used to analyze the difference between the mortality. Significant differences were considered as *p* < 0.05.

## Results

**Fig. 2:**
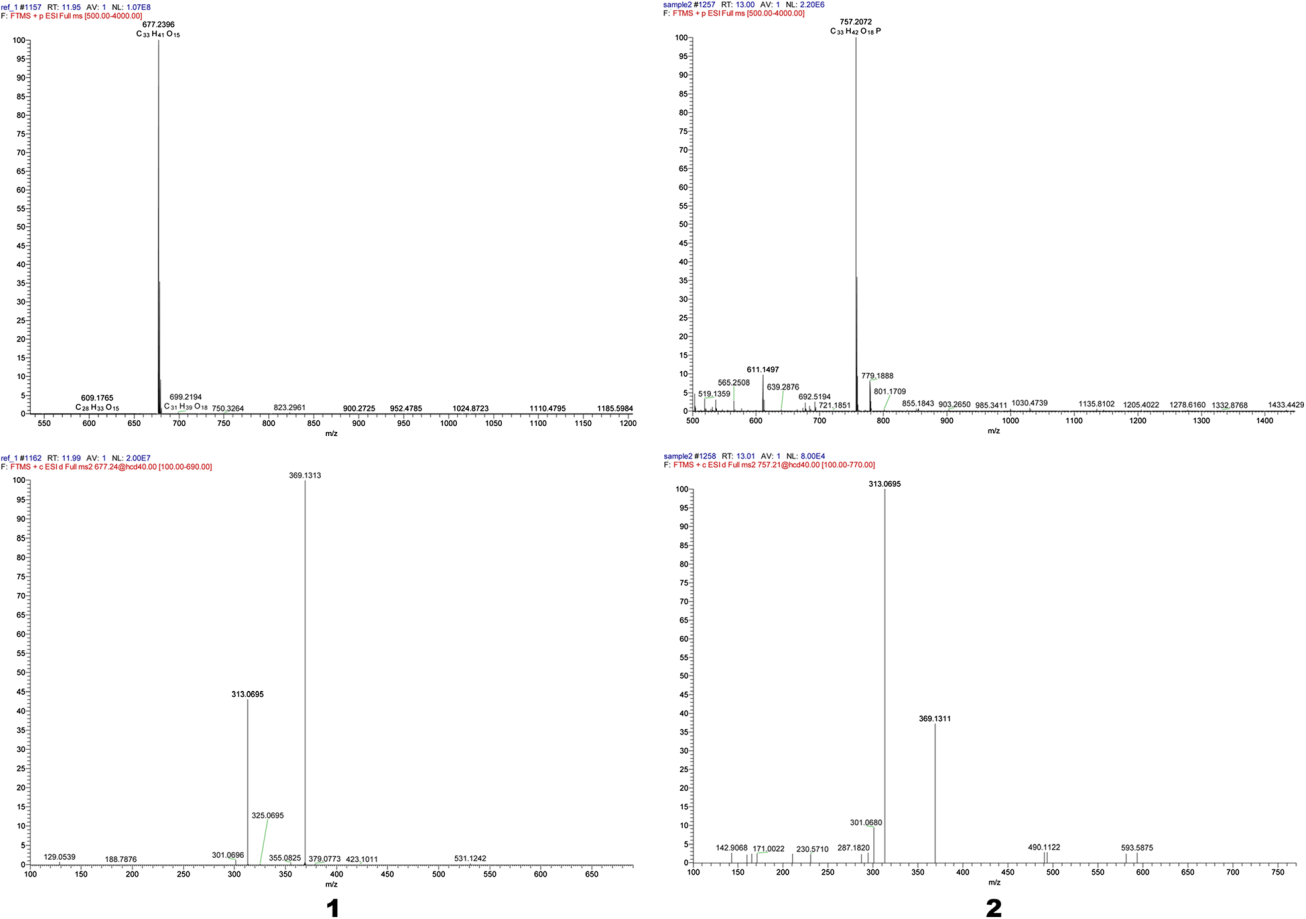
HRESIMS spectrum of compound 1 and 2

Compound **1** was obtained as a yellow, amorphous powder. Its molecular formula (C_33_H_40_O_15_) was established on the basis of high-resolution electrospray ionization mass spectrometry (HRESIMS) (*m/z* 676.7400 [M + H]^+^; calcd for C_33_H_41_O_15_, 677.2396, Fig. 2) in combination with ^13^C nuclearmagnetic resonance (NMR) data (Table 1). The FT-IR spectrum exhibited characteristic absorptions for hydroxy (3396 cm^−1^), carbonyl (1651 cm^−1^), and aromatic (1600 and 1454 cm^−1^) functionalities. It was consistent with the structure of icariin [21].

Compound **2** was obtained as a yellow, amorphous powder. Its molecular formula (C_33_H_42_O_18_ P) was established on the basis of ^13^C NMR and HRESIMS data (*m/z* 757.2034 [M + H]^+^; calcd for C_33_H_42_O_18_P, 757.2072, Fig. 2). The FT-IR spectrum exhibited characteristic absorptions for hydroxy (3384 cm^−1^), carbonyl (1652 cm^−1^), and aromatic (1610 and 1430 cm^−1^) functionalities, it was similar to **1.** Furthermore, **2** also displayed extra absorption peaks at 1160.61 and 918.15 cm^−1^, corresponding to the phosphate group. The ^13^C NMR data (Table 1) of 2 were similar to those of 1, except for the the C-6''' (**1** C-6''':δ_C_61.10; **2** C-6''':δ_C_70.17), it suggested that the phosphate group was located at C-6''', which instead of oxhydryl. Combined with FT-IR, HRESIMS and ^13^C NMR data, suggesting that compound **2** was also a phosphorylated derivative of **1** and the structure was defined as 8-prenylkaempferol-4′-methylether-3-rhamnosyl-7- (6'''-phosphate)-glycoside and given the name 6'''-phosphate icariin.

Table 2 listed the A_570_ values and virus inhibitory rate in the anti-DHAV activity test in DEHs. The A_570_ values of the **1** group ranging from 62.5 μg/mL to 31.25 μg/mL, and the A_570_ values of the **2** group ranging from 312.5 μg/mL to 156.25 μg/mL were significantly higher than that of the VC group. The A_570_ value of the **2** at 156.25 μg/mL was also notably higher than that of the CC group, which indicated that **2** also promote cell growth. The virus inhibitory rates of **2** were higher than that of **1** at similar concentrations, with the highest one at 135.78 %, while the highest of **1** being 98.28 %.

**Table 2:** A_570_ values and virus inhibitory rate in DHAV test on DEHs

Group	Concentration^A^ (μg/mL)	*A*_570_	Virus inhibitory rate(%)	Group	Concentration^A^ (μg/mL)	*A*_570_	Virus inhibitory rate(%)
**1**	250	0.191 ± 0.026^c^	1.72	**2**	1250	0.241 ± 0.026^c^	0.92
	125	0.211 ± 0.032^bc^	18.97		625	0.261 ± 0.010^c^	19.27
	62.5	0.230 ± 0.016^b^	35.34		312.5	0.365 ± 0.032^ab^	114.68
	31.25	0.303 ± 0.013^a^	98.28		156.25	0.388 ± 0.020^a^	135.78
VC		0.189 ± 0.018^c^		VC		0.240 ± 0.014^c^	
CC		0.305 ± 0.006^a^		CC		0.349 ± 0.024^b^	

Data within a column without the same superscripts (a–c) differ significantly (p < 0.05)

^ab^ With ^a^ and ^b^ were no significant differences. ^bc^ With ^b^ and ^c^ were no significant differences

^A^ The safe concentration determined by prior cytotoxicity tests

The mortality of young ducklings in the BC, VC, 1, 2 groups was 0,30,24,21 feathers. All ducklings in the BC group survived. After treatment with **1** and **2**, the young ducklings challenged with DHAV no longer died after 72 h, while all of the ducklings in the VC group died on the 4th day. Both mortality of compound **1** (80 %) and compound **2** (70 %) were lower than that of the VC group (100%), and compound **2** group’s mortality was significantly lower than that of the VC group.

**Fig. 3:**
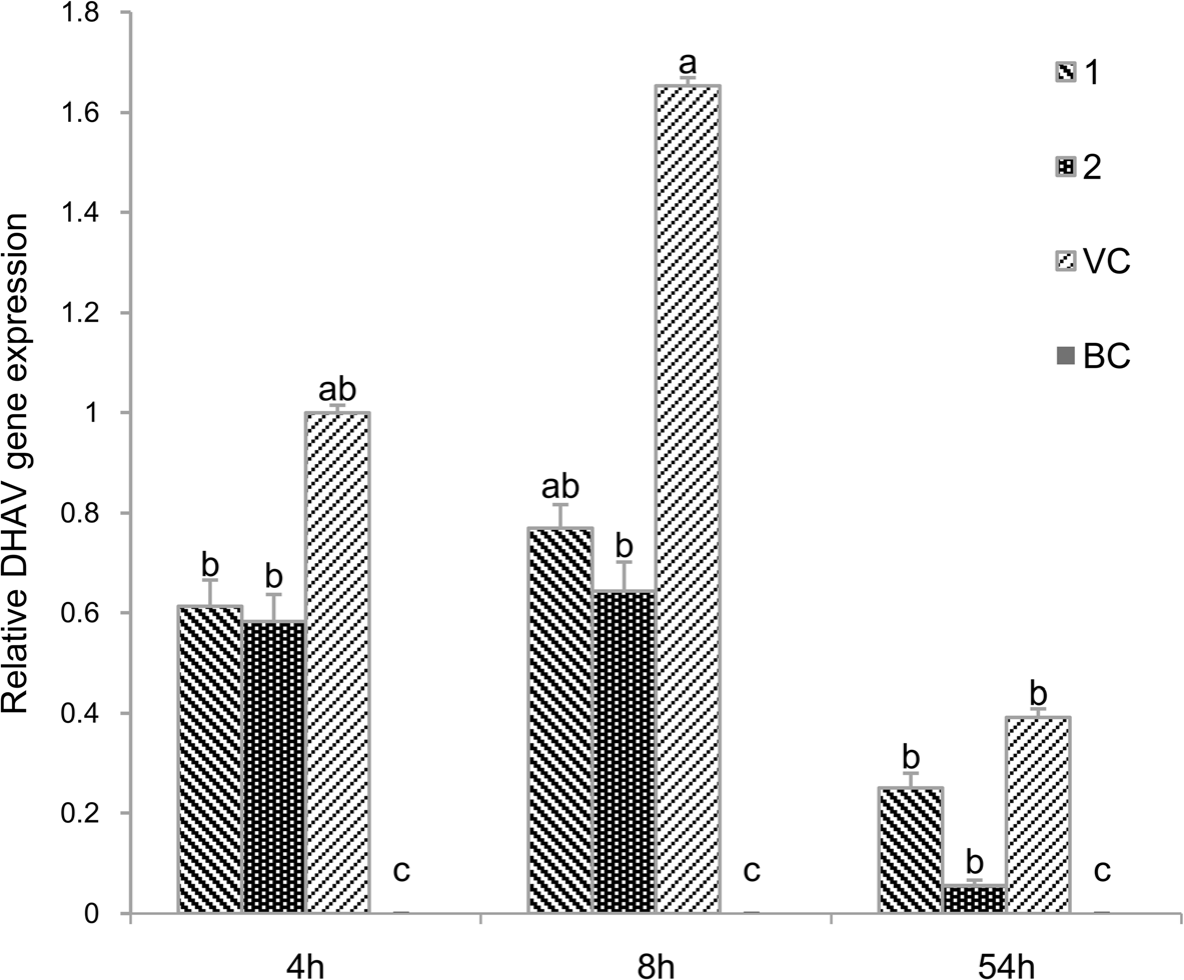
Quantitative analysis of the DHAV gene expression in blood at the 4th, the 8th and the 54th h after injected virus. DHAV gene expression of the VC group at 4 h after injected virus was set to 1 and that of the BC group was set to 0. The different letters on a column differ significantly (*p* < 0.05)

A quantitative analysis of the DHAV gene expression in blood at the 4th, the 8th and the 54th h after injected with virus was illustrated in Fig. 3. No DHAV gene expression was found in the BC group. DHAV gene expression of the VC group in the 4th h after injected virus was set to 1 and that of the BC group was set to 0. The VC group dynamics indicated that the DHAV content first increased and then decreased *in vivo* of ducklings, with the pattern of the **1** and **2** groups similar to that of the VC group. The relative expressions of DHAV gene in blood of the **1** and **2** groups were lower than that of the VC group during the same period. Four hours after DHAV injection, the virus gene expressions in both the **1** and **2** groups were lower than that of the VC group. At the 8th hour after DHAV injection the virus contents of the **1** and **2** groups were lower than that of the VC group with significant difference. At the 54th h after DHAV injection the virus gene content of the **2** group was remarkably lower than that of the VC group.

**Fig. 4:**
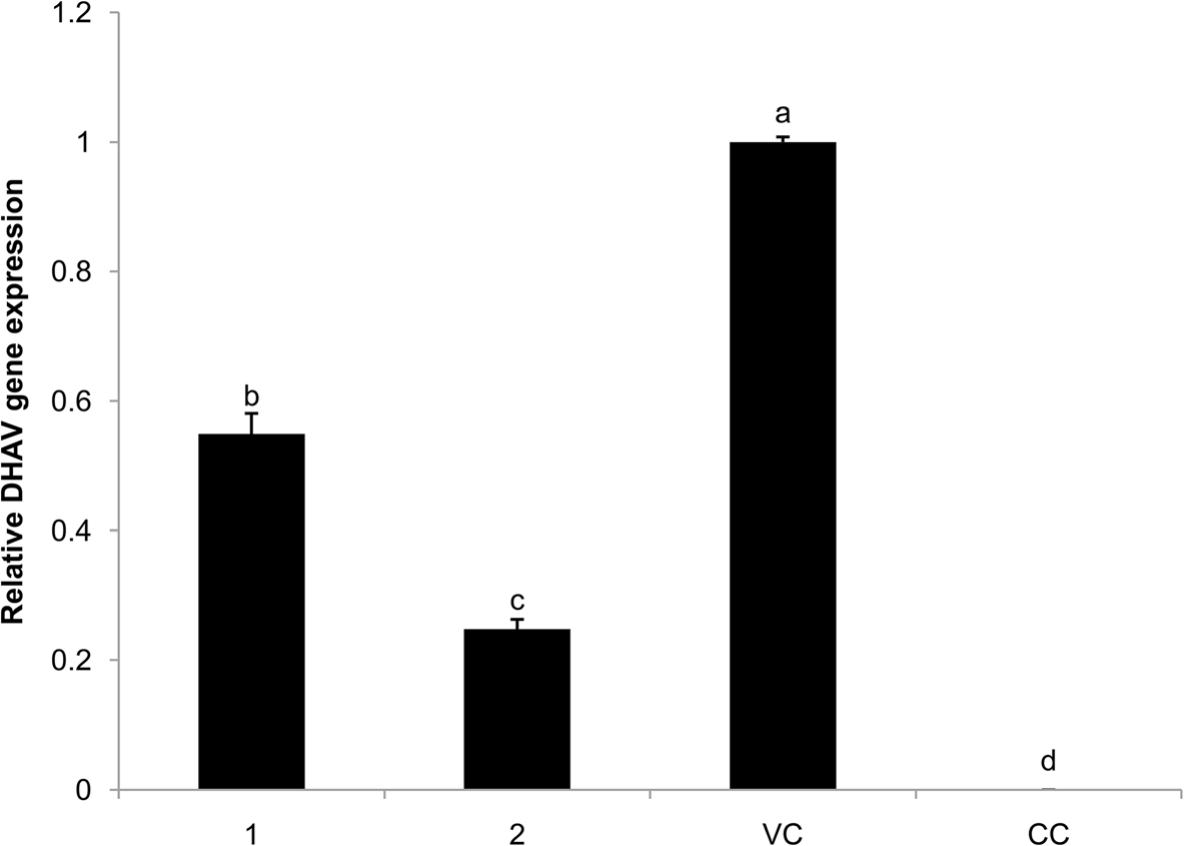
Replication DHAV gene expression in DEHs. Effect of **1** and **2** on DHAV replication. DHAV gene expression of the VC group was set to 1 and that of the BC group was set to 0. The different letters on a column differ significantly (*p* < 0.05)

The effect of **1** and **2** on replication of DHAV was illustrated in Fig. 4. No DHAV gene expression was found in the CC group. DHAV gene expression of the VC group in the twenty-fourth hour was set to 1. Relative DHAV gene expressions of **1** and **2** groups were 0.549 and 0.248; both were significantly lower than that of the VC group. Relative expression of **2** was significantly lower than that of **1**.

**Fig. 5:**
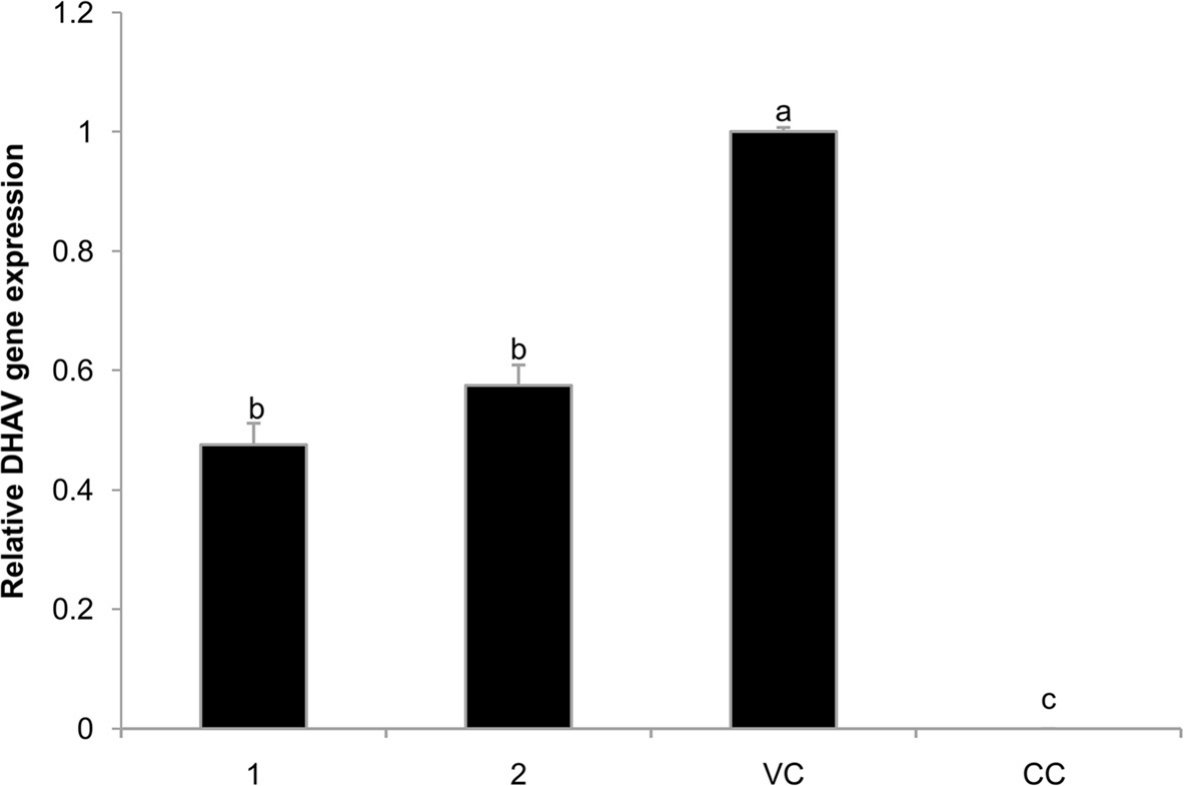
Release DHAV gene expression in DEHs. Effect of **1** and **2** on DHAV release. DHAV gene expression of the VC group was set to 1 and that of the BC group was set to 0. The different letters on a column differ significantly (*p* < 0.05)

The effect of **1** and **2** on the release of DHAV was illustrated in Fig. 5. No DHAV gene expression was found in the CC group. The DHAV gene expression of the VC group in the 48 h was set to 1 and that of the BC group was set to 0. Relative expressions of **1** and **2** were 0.476 and 0.575. And both were significantly lower than that of the VC group.

## Discussion

Acid ions cannot only bind to virus or cell skin cation to inhibit viral invasion or replication, but also enhance the antiviral effect and water solubility of drugs [22]. This experiment sought for the optimal conditions for phosphorylated modification of **1** and successfully introduced phosphate group. It still preserved the flavones’ basic chemical structure. The results showed that **2** had significantly better water solubility than **1** and was suitable for practical application. The higher the drug’s maximum concentration was, the safer it was inside the cell *in vitro* [23]. In the present experiment, the maximum tolerable dose of **2** (1250 μg/mL) was much higher than that of **1** (250 μg/mL). The results indicated that the cellular toxicity of **1** may be significantly reduced by phosphorylation.

The antiviral activity assays of **1** and **2***in vitro* using MTT method indicated that higher A_570_ values were associated with improved antiviral properties [24]. Virus inhibitory rates directly reflected the drug’s antiviral performance. Within a certain range, the A_570_ values of **1** and **2** were significantly higher than that of the VC group; with some A_570_ values of **2** even higher than the CC group. Within the effective concentration range, the virus inhibitory rates of **2** and the maximum inhibitory rate were both higher than those of **1**. The results suggested that both **2** and **1***in vitro* possessed strong anti-DHAV function, with **2** stronger than **1**.

In clinical trials, DHAV was infectious for young ducklings aged up to 3 weeks, particularly those less than 1-week-old, whose mortality rate was higher than 90 % [25]. Therefore, we selected 3-day-old young ducklings for this experiment taking into account the clinical significance. In this study, all ducklings in the BC group survived. After treatment with **1** and **2**, the young ducklings challenged with DHAV no longer died after 72 h, while all of the ducklings in the VC group died on the 4th day. Mortalities of the **1** and **2** groups were both lower than that of the VC group, which proved that both **1** and **2** had anti-DHV effect. If focused on the mortality of the **2** group, we could find that it was significantly lower than that of the VC group, indicating its superior effect compared to **1**. The results were consistent with the anti-DHAV invading cells, *in vitro*.

DHAV is classified in the family *parvoviridae* [26]. It completes a cycle of replication in 6–8 h, *in vivo*. In early phase of infection, humoral immunity to anti-DHAV is still not active. Instead, organisms mainly relied on the direct antivirus effects of the drug. A quantitative analysis of the DHAV gene expression in blood at the 4th, the 8th and the 54^th^h after injected with virus was illustrated in Fig. 3. Four hours after virus invasion, it was possible that virus still failed to complete replication. As the gene expressions of DHAV in the **1** and **2** groups were lower than that of VC group, no substantial differences between virus level in blood of the **1**, **2** and VC groups was observed. At the 8^th^h, the virus had at least completed one replication cycle. The blood virus contents in the **1** and **2** groups were significantly lower than that of the VC group. In addition, since **2** was more potent, the **2** group blood virus content was lower than that of the **1** group. At the 54^th^h, with the activation of humoral immunity and increased cytokine levels, the levels of the **1**, **2** and VC groups’ blood virus plummeted. The **1** and **2** groups’ blood virus contents were lower than that of the VC group and this is especially significant for the **2** group. The results were consistent with the death of the ducklings in the challenging experiment. We are submitting another article has reported that the contents of IFN-γ, IL-2 and IL-6 in the **1** and **2** groups were significantly higher than those in the VC group at the 54^th^h, which indicated that **1** and **2** could obviously improve immunomodulatory activity. It also demonstrated that the treatment of traditional Chinese medicine maybe more reflected in the balance of the internal systems, rather than directly inhibited or killed pathogenic microorganisms.

Previous studies established a solid basis for the molecular detection of DHAV using Real-time PCR [19]. RT-PCR with its higher specificity, less PCR contamination and higher automation has been widely applied in the field of diagnosis and detection of DHAV. During virus replication, the relative quantitative gene expressions of the **1** and **2** groups were significantly lower than that of the VC group (Fig. 4). The results suggested that **1** and **2** greatly inhibited the replication of DEHs *in vitro*, and that **2** outperformed **1**. Lu et al. [22] also found that acid ions prevented virus from replicating inside cells. Acid ions of PO_4_^3−^ introduced into **1** enhanced the inhibition of DHAV *in vitro*.

In virus release phase (Fig. 5), the relative quantitative gene expressions of the **1** and **2** groups were significantly lower than that of the VC group. Both had similar inhibitory effect on DHAV releasing DEHs *in vitro*. Therefore, based on the results of virus replication and release *in vitro*, both **1** and **2** had significant inhibitory function on virus activity, although the **2** was better due to its acidic groups. Due to direct inhibition of virus replication in the **1** and **2** groups, and consequent lowered blood viral content, the ducks were spared from possible harm and showed a decreased Mortality, especially true for the **2** group. The antiviral immune response stimulated by the **1** and **2** also helped in substantially reducing the damage due to DHAV. Additional studies to confirm our findings *in vivo* are required.

### Supporting information

^13^C NMR of compound **1**and compound **2**.

## Conclusion

The compound **2** was also a phosphorylated derivative of **1** and the structure was defined by FT-IR, HRESIMS and ^13^C NMR. Compound **1** and **2** effectively resisted DHAV from invading DEHs *in vitro* , and had good therapeutic effects in young ducklings infected with DHAV. The Mortality was significantly decreased and **2** showed better effect than **1**, it showed that the novel structure was worth further research for application. The mechanism underlying the antiviral activity of **2** and **1***in vitro* was probably mediated by interference with the replication and release of virus. Due to the greater inhibition on virus replication than **1**, **2** showed better antiviral effect.

## References

[1] Levine PP, Fabricant J (1950). A hitherto-undescribed virus disease of ducks in North America. Cornell Vet.

[2] Woolcock PR (2003). Diseases of Poultry.

[3] Pan M, Yang X, Zhou L, Ge X, Guo X, Liu J, Zhang D, Yang H (2012). Duck Hepatitis A Virus Possesses a Distinct Type IV Internal Ribosome Entry Site Element of Picornavirus. J Virol.

[4] Reaiche GY, Le Mire MF, Mason WS, Jilbert AR (2010). The persistence in the liver of residual duck hepatitis B virus covalently closed circular DNA is not dependent upon new viral DNA synthesis. Virology.

[5] Zhang YQ, Sun ZA, Liu JG, Wang DY, Zhang BK, Yi F, Fan YP, Liu D, Liu X, Liu C (2012). Flavone ingredients can synergistically inhibit NDV infecting cell and improve ND vaccine’s protective rate. Int J Biol Macromol.

[6] Havsteen BH (2002). The biochemistry and medical significance of the flavonoids. Pharmacol Ther.

[7] Kong XF, Hu YL, Rui R, Wang DY, Li XG (2004). Effects of Chinese herbal medicinal ingredients on peripheral lymphocyte proliferation and serum antibody titer after vaccination in chicken. Int Immunopharmacol.

[8] H-f L, X-y G, Yang W-z, K-d L, Ye M, Sun C, Lu S, Guo D-a (2012). Antioxidant flavonoids from Epimedium wushanense. Fitoterapia.

[9] Zhang DW, Cheng Y, Wang NL, Zhang JC, Yang MS, Yao XS (2008). Effects of total flavonoids and flavonol glycosides from Epimedium koreanum Nakai on the proliferation and differentiation of primary osteoblasts. Phytomedicine.

[10] Wu H, Lien EJ, Lien LL (2003). Chemical and pharmacological investigations of Epimedium species: a survey. Progress in drug research Fortschritte der Arzneimittelforschung Progres des recherches pharmaceutiques.

[11] Wang Y, Dong H, Zhu M, Ou Y, Zhang J, Luo H, Luo R, Wu J, Mao M, Liu X (2010). Icariin exterts negative effects on human gastric cancer cell invasion and migration by vasodilator-stimulated phosphoprotein via Rac1 pathway. Eur J Pharmacol.

[12] Sun Y, Wang J, Luo Y (2002). Effects of total flavones from Epimedium L. on IL-2 and NK activity in immunodepressant mice. Chinese Traditional and Herbal Drugs.

[13] Xiong W, Chen Y, Wang Y, Liu JG (2014). Roles of the antioxidant properties of icariin and its phosphorylated derivative in the protection against duck virus hepatitis. BMC Vet Res.

[14] Sauerbrei A, Schacke M, Schultz U, Egerer R, Merkle I, Glebe D, Gerlich W, Wutzler P (2005). Alternative methods for validation of cell culture infection with duck hepatitis B virus. J Virol Methods.

[15] Schacke M, Glueck B, Wutzler P, Sauerbrei A (2009). In vitro cultivation and cryopreservation of duck embryonic hepatocytes. J Virol Methods.

[16] Biacchesi S, Skiadopoulos MH, Yang LJ, Murphy BR, Collins PL, Buchholz UJ (2005). Rapid human metapneumovirus microneutralization assay based on green fluorescent protein expression. J Virol Methods.

[17] Kaneko H, Fujiwara T, Mori S, Shigeta S (2000). Evaluation of antiviral agents for adenovirus using the MTT method in vitro. Nippon Ganka Gakkai Zasshi.

[18] Takeuchi H, Baba M, Shigeta S (1991). An application of tetrazolium (MTT) colorimetric assay for the screening of anti-herpes simplex virus compounds. J Virol Methods.

[19] Yang M, Cheng A, Wang M, Xing H (2008). Development and application of a one-step real-time Taqman RT-PCR assay for detection of Duck hepatitis virus type1. J Virol Methods.

[20] Livak KJ, Schmittgen TD (2001). Analysis of relative gene expression data using real-time quantitative PCR and the 2(T)(−Delta Delta C) method. Methods.

[21] Xia Q, Xu D, Huang Z, Liu J, Wang X, Wang X, Liu S (2010). Preparation of icariside II from icariin by enzymatic hydrolysis method. Fitoterapia.

[22] Lu Y, Wang D, Hu Y, Huang X, Wang J (2008). Sulfated modification of epimedium polysaccharide and effects of the modifiers on cellular infectivity of IBDV. Carbohydr Polym.

[23] Verma A, Prasad KN, Singh AK, Nyati KK, Gupta RK, Paliwal VK (2010). Evaluation of the MTT lymphocyte proliferation assay for the diagnosis of neurocysticercosis. J Microbiol Methods.

[24] Huang X, Wang D, Hu Y, Lu Y, Guo Z, Kong X, Sun J (2008). Effect of sulfated astragalus polysaccharide on cellular infectivity of infectious bursal disease virus. Int J Biol Macromol.

[25] Tseng C-H, Knowles NJ, Tsai H-J (2007). Molecular analysis of duck hepatitis virus type 1 indicates that it should be assigned to a new genus. Virus Res.

[26] Kim M-C, Kwon Y-K, Joh S-J, Lindberg AM, Kwon J-H, Kim J-H, Kim S-J (2006). Molecular analysis of duck hepatitis virus type 1 reveals a novel lineage close to the genus Parechovirus in the family Picornaviridae. J Gen Virol.

